# The importance of nonlinear tissue modelling in finite element simulations of infant head impacts

**DOI:** 10.1007/s10237-016-0855-5

**Published:** 2016-11-21

**Authors:** Xiaogai Li, Håkan Sandler, Svein Kleiven

**Affiliations:** 1 Division of Neuronic Engineering, School of Technology and Health, Royal Institute of Technology—KTH, 141 52 Huddinge, Sweden; 2 Department of Surgical Sciences/Forensic Medicine, Uppsala University, Uppsala, Sweden; 3 National Board of Forensic Medicine, Uppsala, Sweden

**Keywords:** Infant skull fracture, Finite element head models, Head injury, Impact angle influence, Nonlinear tissue modelling

## Abstract

Despite recent efforts on the development of finite element (FE) head models of infants, a model capable of capturing head responses under various impact scenarios has not been reported. This is hypothesized partially attributed to the use of simplified linear elastic models for soft tissues of suture, scalp and dura. Orthotropic elastic constants are yet to be determined to incorporate the direction-specific material properties of infant cranial bone due to grain fibres radiating from the ossification centres. We report here on our efforts in advancing the above-mentioned aspects in material modelling in infant head and further incorporate them into subject-specific FE head models of a newborn, 5- and 9-month-old infant. Each model is subjected to five impact tests (forehead, occiput, vertex, right and left parietal impacts) and two compression tests. The predicted global head impact responses of the acceleration–time impact curves and the force–deflection compression curves for different age groups agree well with the experimental data reported in the literature. In particular, the newly developed Ogden hyperelastic model for suture, together with the nonlinear modelling of scalp and dura mater, enables the models to achieve more realistic impact performance compared with linear elastic models. The proposed approach for obtaining age-dependent skull bone orthotropic material constants counts both an increase in stiffness and decrease in anisotropy in the skull bone—two essential biological growth parameters during early infancy. The profound deformation of infant head causes a large stretch at the interfaces between the skull bones and the suture, suggesting that infant skull fractures are likely to initiate from the interfaces; the impact angle has a profound influence on global head impact responses and the skull injury metrics for certain impact locations, especially true for a parietal impact.

**Electronic supplementary material:**

The online version of this article (doi:10.1007/s10237-016-0855-5) contains supplementary material, which is available to authorized users.

## Introduction

Skull fractures, especially multiple fractures, bilateral fractures, and fractures with complex configuration, are often suspicious for child abuse (Meservy et al. 1987; Flaherty et al. 2014). However, accidental household short falls can also cause skull fractures (Gruskin and Schutzman 1999) although such falls rarely cause severe or fatal brain injury (Chadwick et al. 2008). Distinguishing whether the observed skull fractures were caused by an accidental fall or abuse is still very challenging and has attracted substantial attention in the forensic community (Bilo et al. 2010; Ehsani et al. 2010; Hamel et al. 2013; Holck 2005; Hymel et al. 2013; Jenny et al. 2014). Epidemiological studies have been used as a tool to aid forensic investigators on the diagnosis of suspected abuse (Ehsani et al. 2010). However, inferring the risk of injuries based on epidemiological studies is logically flawed as many factors influence the severity of injury, e.g. age, head impact location and impact surface (Hamel et al. 2013). A reliable tool providing scientific evidences for case-specific investigation is needed in the era of evidence-based medicine.

To understand the aetiology of infant skull fracture due to falls, Weber (1984, 1985) conducted drop tests with whole-body child post-mortem human subjects (PMHS) from a height of 82 cm impacted at the parieto-occipital region onto surfaces of varying stiffness. All drops onto stiff tile floor resulted in simple linear fractures. Weber’s study, being the first systematic study of fracture mechanisms in infant cadavers, provides valuable information for forensic investigations regarding infant skull fracture. However, the lack of quantitative measurements limits its use in validating biomechanical models. Prange et al. (2004) reported three newborn head drop tests from 15 and 30 cm height impacted at five different locations (forehead, occiput, vertex, right and left parietal region). Using more specimens, including the three newborns documented in Prange et al. (2004), Loyd (2011) reported drop tests with six newborns, one 1-, 5-, 9-, 11- and 22-month-old, as well as 9- and 16-year-old specimens. More importantly, the actual impact angles were reported for many drop tests, together with the acceleration–time impact curves. The force–deflection curves for the whole head compression tests were also measured and depicted. Some of the force–deflection curves were published in a more recent study (Loyd et al. 2015). The impact and compression tests reported in Loyd (2011) and Prange et al. (2004) have since been used as the primary source for validating biomechanical infant head models. Considering ethical concerns and limited availability of human specimens, experimental studies using infant porcine specimens have also been performed to help understand infant skull fracture patterns and mechanisms (Deland et al. 2016; Powell et al. 2012, 2013).

Parallel to the experimental efforts, several studies have developed infant FE head models and the model predictions were compared with the aforementioned experiments. Coats et al. (2007) developed a 5-week-old infant head model and studied the relative importance of brain material properties and anatomical variations in infant suture and scalp tissue. The model also predicted that infant linear skull fractures may occur with head-first fall heights of 82 cm onto concrete, as shown in Weber (1984). Roth et al. (2010) constructed a model based on a 17-day-old specimen, and the model predictions were compared with the drop and compression tests by Prange et al. (2004). Recently, Li et al. (2015a) developed statistical skull geometry model from 0- to 3-year-olds based on head CT head scans from 56 children, including the ones from 0- to 3-month-olds presented in an earlier study (Li et al. 2013a). Based on the obtained geometrical information on the head and suture sizes, infant FE models of a newborn (Li et al. 2013a), 6-month-olds (2013b, 2016), as well as other ages from 0- to 9-month-olds were developed (Li et al. 2015b), morphed from a baseline FE mesh of a 6-month-old.

Despite the aforementioned modelling efforts, a model capable of capturing head responses under various impact scenarios has not been reported. This is hypothesized partially attributed to the use of simplified linear elastic models for the suture, scalp, and dura mater (see detailed information in Sect. 2.2). Further, infant skull bones are often simplified as isotropic material (Li et al. 2013a, b, 2015a, b, 2016; Roth et al. 2010), despite the fact that the grain fibre patterns are clearly visible to naked eyes in newborns (Coats and Margulies 2006), causing a much larger stiffness in the fibre direction confirmed by mechanical tests (Kriewall 1982; McPherson and Kriewall 1980). In addition, the performance of the developed models is usually assessed at a limited number of impact locations, e.g. occiput impact (Coats et al. 2007), forehead and parietal impact (Li et al. 2013b), or consider only the peak value instead of the entire acceleration–time impact curve (Li et al. 2013a). Moreover, it is not mentioned whether or not the actual impact angles were used as the experiments when validating the head models in previous studies. The problem is further complicated as the impact angles for the three newborn drop tests were not provided in neither Prange et al. (2004) nor Loyd (2011), making it impossible to use the same impact angle for the studies that chose to compare with these experimental data. Yet, it is unknown whether or how the impact angle affects the head response.

We hypothesized that experimentally based nonlinear elasticity of the soft tissues of infant head models, together with accurate reproduction of the impact angle, would improve the correlation with experimental drop tests. For this, a new Ogden hyperplastic model for suture is developed; a new approach to include both the increase in stiffness and decrease in anisotropy with age in the infant skull bone is proposed. Subject-specific FE models of infant heads of a newborn, 5-month-olds (5 M) and 9-month-olds (9 M) are developed incorporating the nonlinear elastic models for suture, scalp and dura mater, as well as orthotropic skull bone. The models are subjected to extensive drop tests with the same impact angle as in the experiments, as well as compression tests. The influence of impact angle is then studied, followed by a parametric study using linear elastic models for the soft tissues, and with the sutures removed.

## Methods

### FE model generation

**Fig. 1:**
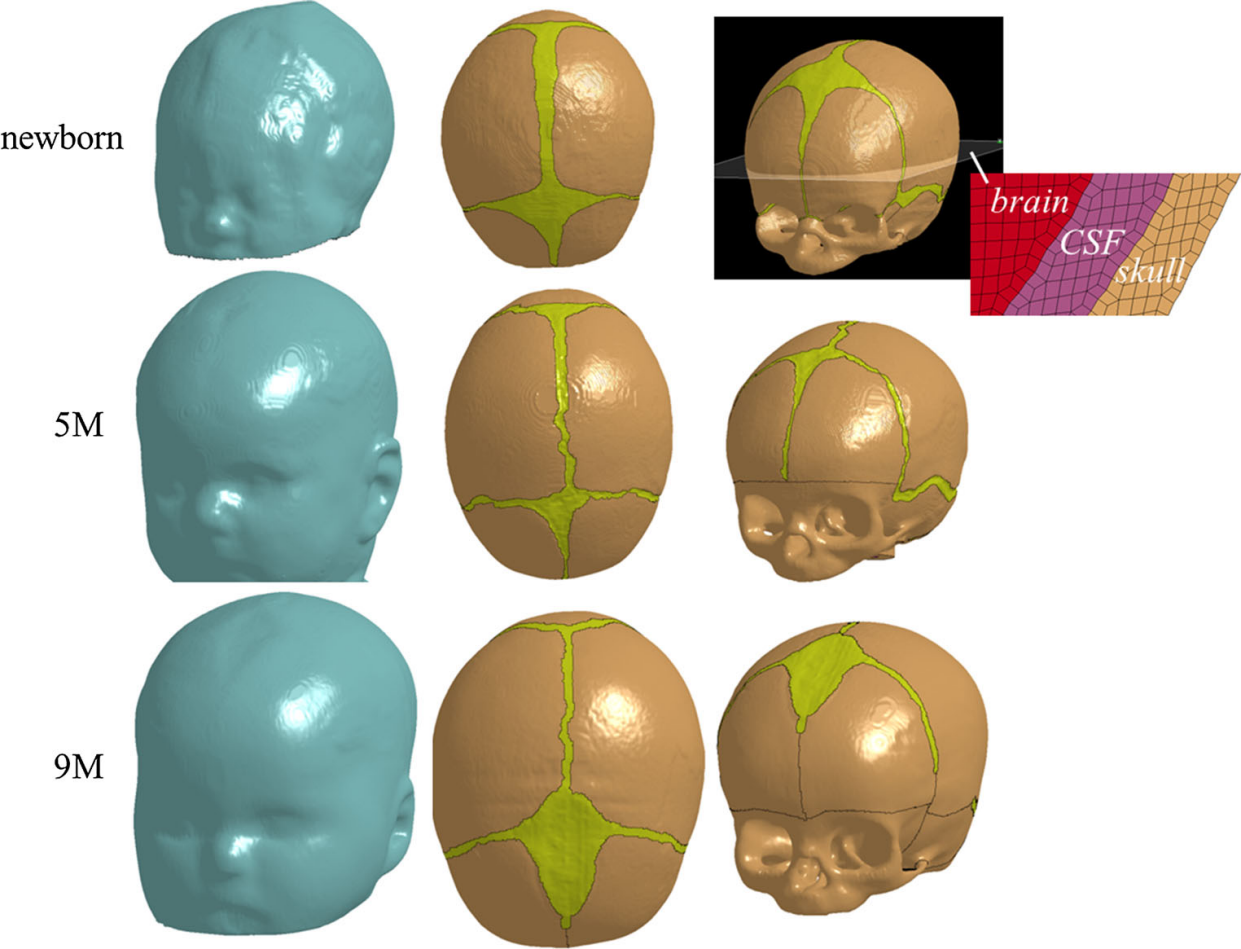
Generated FE models of the newborn (*upper row*), 5 M (*middle row*) and 9 M heads (*lower row*). The skull is composed of bony plates connected by sutures and fontanelles. To improve the illustration, the FE meshes are made invisible and are only illustrated in the enlarged image of the newborn model

The FE meshes are generated based on the geometrical reconstructions of computerized tomography (CT) images from Uppsala University Hospital, which were subjected to postmortal forensic investigations at the National Board of Forensic medicine, Uppsala, for various reasons that are not related to the present study. The use of these anonymized CT images was approved by the local ethical committee. No structural abnormalities are observed in the CT images. The resolution for all the CT images was $0.49 \times 0.49 \times 0.625\,\hbox {mm}^{3}$. The scalp, skull, cerebrospinal fluid (CSF), brain and sutures are segmented semi-automatically using the software Slicer 3D (Pieper et al. 2004). Initial segmentation is done by thresholding. Manual segmentation is then used to delineate regions that are visible but could not be extracted (e.g. the suture). Three-dimensional triangular surface meshes are thereafter generated based on the segmented images and serve as input to the software Hexotic to generate hexahedron elements using an octree algorithm (Maréchal 2009). The total number of the elements in the newborn, 5 and 9 M models is 3.8, 3.6 and 5.3 million, respectively. The typical element size in the skull is about 0.4 mm (Fig. 1). The jaw bones are removed from the heads to replicate the experiments reported in Loyd (2011) that the developed models are compared with. The FE meshes are then scaled to match the dimensions of the corresponding cadaveric heads using characteristic length (CL) (sum of head length, head width and head circumference) (see “Appendix 1” for details), since both impact acceleration and compression stiffness are dependent on head size, according to Melvin (1995). The amount of lower scalp elements included in the drop simulations are then adjusted until the head mass in the model becomes the same as in the experiment (Fig. 10 illustrates the final selected scalp elements that are included in the drop simulations). The anatomical features of the generated FE models including the head sizes, scalp and suture thicknesses are reported in “Appendix 1”. All simulations are conducted with LS-Dyna 971 using an explicit dynamic solving method.

### Material modelling for suture, scalp and dura

Soft connective tissues behave nonlinearly in response to external forces, including the suture, scalp and dura mater (Herring and Ochareon 2005; Jasinoski et al. 2010; Jaslow 1990), with collagen as the main load-carrying element in a wide variety of soft tissues. Under stretch, a typical uniaxial stress–strain curve of soft tissue starts with a toe region, then enters a linear region and finally reduces in slope as the tissue yields and fails (Holzapfel 2001; Meyers et al. 2008; Winkelstein 2012). The toe region represents “un-crimping” of the wavy collagen fibrils, and this part of the curve shows a relatively low stiffness. As the crimp is removed, more stretched fibres become fully engaged against the mechanical load, and the tissue enters the linear region based on which the Young’s modulus is calculated. Although Young’s modulus is designed to describe the stiffness of a perfectly elastic material, it is often reported for soft tissues as well by calculating the gradient of the stress–strain curves measured directly after the toe region in the linear region (McKee et al. 2011). To capture the nonlinear elastic behaviour of the tissue, more advanced models are needed (Fung 1967; McKee et al. 2011). In the following sections, the development of a new Ogden model for suture is presented, followed by the nonlinear material modelling for scalp and dura adopted in the infant head models.

#### Suture modelling

**Fig. 2:**
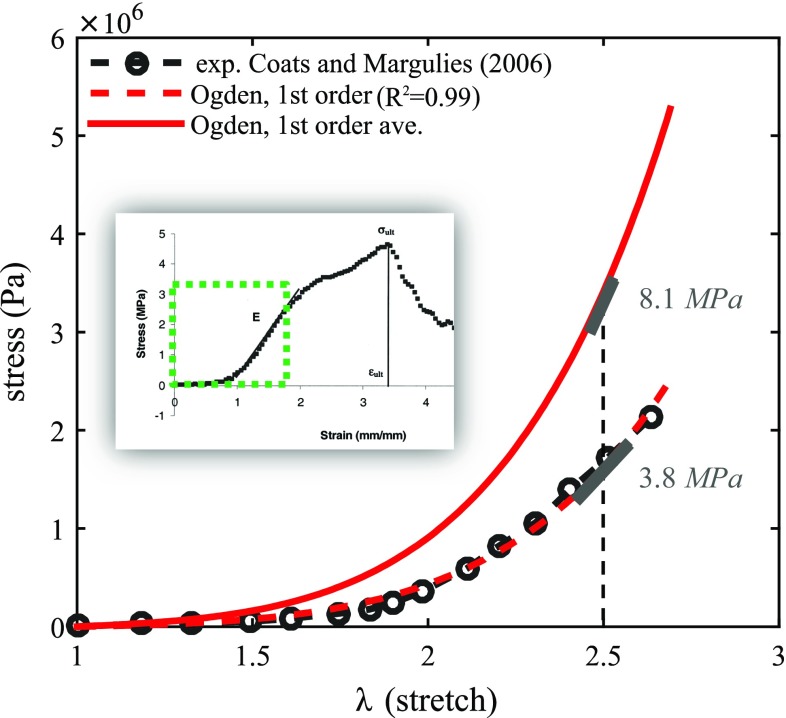
Fitting of uniaxial experimental data for suture. The entire experimental stress–strain curve from zero strain, indicated by the dashed-green frame, is used to fit the first-order Ogden model with strain being converted to stretch ratio. The fitted model results in Young’s modulus of 3.8 MPa calculated at $\lambda =2.5$, approximately the same as in the experiment. $\mu _1$ is then scaled up to obtain Young’s modulus of 8.1 MPa at $\lambda =2.5$, to represent the average elastic modulus for all the tested specimens

Coats and Margulies (2006) performed uniaxial tension tests using 14 suture specimens from 11 infant calvaria (21-week gestation to 12 months old), including a 2-month-old donor. The entire stress–strain curve for one specimen from the 2-month-olds was presented, while for others, Young’s modulus was calculated as the gradient from the linear region (Fig. 2). The mean Young’s modulus across all age groups was 8.1 MPa. Being the first, and still the only experimental study on human specimens at loading rate similar to low height impact, the reported Young’s modulus, since then, has been widely used for linear elastic suture modelling in infant head models (Coats et al. 2007; Li et al. 2013b, 2016; Roth et al. 2010).

To account for the large deformation nonlinear elasticity, in this study, a first-order Ogden hyperelastic model is fitted to the uniaxial tension stress–strain curve from Coats and Margulies (2006) via an iterative least-square algorithm of Levenberg–Marquardt (MATLAB) according to:

1 $$\begin{aligned} \hbox {Nominal stress}=\mu _1 \left( {\lambda ^{-1+\alpha }-\lambda ^{-1-\frac{\alpha _1 }{2}}} \right) \end{aligned}$$

where $\lambda$ is the stretch in the uniaxial direction, and $\mu _{1},\,\alpha _1$ are Ogden constants. The fitted Ogden model describes well the entire experimental stress–stretch curve of the suture (coefficient of determination $R^{2}=0.99$), including the initial toe region and the linear elastic region (Fig. 2). As mentioned earlier, of all the 14 tested specimens, only one stress–strain curve for a 2-month-old was reported and is fitted. Given that the Young’s modulus is not significantly affected by donor age (Coats and Margulies 2006), the obtained value of $\mu _1$ is then scaled up to represent the average Young’s modulus (8.1 MPa) for all the tested specimens (Fig. 2). Hence, the same set of Ogden constants is used in all the three models (Table 1).

**Table 1:** Summary of material properties used for the infant head models

	Skull bone parameters for the different age groups					
	$\hbox {Newborn}^{\mathrm{a}}$		5 M		9 M	
	Parietal	Occipital	Parietal	Occipital	Parietal	Occipital
$\rho ^{b}$	2150.0	2150.0	2080.0	2080.0	2075.3	2075.3
$E_1\,(\hbox {MPa})$	731.7	555.5	849.3	650.9	878.7	686.8
$E_2 =E_3\,(\hbox {MPa})$	266.2	211.2	528.6	392.9	615.9	474.6
$G_{23}\,(\hbox {MPa})$	111.8	88.7	222.1	165.1	258.7	199.4
$G_{12} =G_{31}\,(\hbox {MPa})$	194.8	150.8	285.5	215.9	310.6	241.3

Material parameters for other tissues in the head						
Tissue	Material constants	Density ($\hbox {kg}{/}\hbox {m}^{3})$	Poisson’s ratio	References		
Brain	$\mu _{1}=53.8\,\hbox {Pa}$, $\alpha _{1}=10.1$, $\mu _{2}=-120.4\,\hbox {Pa}$, $\alpha _{2}=-12.9$	1040.0	${\sim }$0.5	Kleiven (2007)		
CSF	$K=2.1\,\hbox {GPa}$	1000.0	0.5	Kleiven (2007)		
Suture	$\mu _{1}=1.48\times 10^{4}\,\hbox {Pa}$, $\alpha _{1}=6.9$	1133.0	0.499	This study		
Scalp outer	$\mu _{1}=1.30\times 10^{4}\,\hbox {Pa}$, $\alpha _{1}=24.2$	1133.0	${\sim }$0.5	Fahlstedt et al. (2015)		
Scalp inner	$\mu _{1}=3.99\times 10^{3}\,\hbox {Pa}$, $\alpha _{1}= 8.8$	1133.0	${\sim }$0.5	Fahlstedt et al. (2015)		
Dura mater	Mooney–Rivlin model $C_{1}= 1.18\,\hbox {MPa}$, $C_{2}=0.295\,\hbox {MPa}$	1133.0	0.49	Bylski et al. (1986)		

$^{\mathrm{a}}$The anisotropy ratio and skull bone stiffness for the newborn model are calculated corresponding to a 34-week gestation (i.e. ${-}$1.35 month) consistent with the age of the specimen in the experiments in Loyd (2011), which the developed model to is compare with

$^{\mathrm{b}}$The skull bone density is obtained by linearly extrapolating the full-term skull density to the adult skull density over 18 years as in Loyd (2011)

#### Scalp modelling

Linear elastic model is widely used for scalp modelling in FE head models of adult with Young’s modulus of 16.7 MPa originally derived from adult Monkey (Galford and McElhaney 1970). Infant head models often adopt the same Young’s modulus for scalp (Coats et al. 2007; Li et al. 2013b, 2016; Roth et al. 2010). Recently, Fahlstedt et al. (2015) presented an improved two-layered scalp model incorporating hyperplastic and viscoelastic behaviour of scalp, superior in producing realistic performance under impact loading than linear elastic scalp model. Similarly, in this study the scalp in the infants is modelled with two layers, representing a dense connective tissue layer and an adipose tissue layer. Both layers are modelled with a first-order Ogden hyperplastic model, with the parameters adjusted from Fahlstedt et al. (2015) in the head model of an adult. The adipose tissue mainly contains fat and is softer. Due to the lack of paediatric data, the same material constants for adipose tissue are assumed in the infant as in the adults. The outer layer of scalp (scalp skin) is scaled to be 1/10th of the adult value, which is an assumption to account for a softer scalp skin in infants felt by palpation. In vivo tests using volar forearm skin from subjects aged between 6 months and 90 years did show human skin stiffness increases with age (Diridollou et al. 2001). Nevertheless, a parametric study of scalp material constants shows minimal changes in the acceleration–time curve (see “Appendix 2” for details). Considering the minimal changes with a factor of 1/10th for the scalp skin, one may choose the same material properties as adults until experimental data on infant scalp are available. The parameters for the infant scalp used in the models are listed in Table 1.

#### Dura mater modelling

Linear elastic model with Young’s modulus of 31.5 MPa is commonly used in infant head models (e.g. Roth et al. 2010; Li et al. 2013b, 2016) adopted from adult FE head models, originally derived from a tension test by Galford and McElhaney (1970) of human dura. In this study, a Mooney–Rivlin hyperelastic model is used for dura mater, with the parameters determined from experimental tests of foetal dura mater reported in Bylski et al. (1986) (Table 1). Four-node membrane elements with a thickness of 0.5 mm are used, as measured in the same study.

### Skull bone modelling

Young’s modulus of infant skull bones in both the parallel- and perpendicular-to-fibre directions ($$E_1 ,E_2)$$ was measured by Kriewall (1982) using 554 specimens from 16 foetal calvaria (20- 42-week gestation), including the ones presented earlier by McPherson and Kriewall (1980). However, the tests were performed at low rates not applicable for impact study. While Coats and Margulies (2006) measured the Young’s modulus of infant skull bones from 23 calvaria (21-week gestation to 13 months old) under high strain rate suitable for use in low height impact, only the Young’s modulus along the perpendicular direction ($$E_2$$) was measured due to the limited availability of specimens. To overcome the lack of $$E_1$$, Coats et al. (2007) used a scaling approach and obtained $$E_1$$ by multiplying $$E_2$$ from Coats and Margulies (2006) with an anisotropy ratio based on direction-specific skull bone data from McPherson and Kriewall (1980), assuming that anisotropy ratio ($$E_1 /E_2)$$ remains the same at different strain rates. The scaling approach allowed incorporating mechanical effect of grain fibres in the skull of a 5-week-old infant head model (Coats et al. 2007), while other studies chose to simplify infant skull modelling as an isotropic material (Li et al. 2013a, b, 2015a, b, 2016; Roth et al. 2010), taking $$E_2$$ from Coats and Margulies (2006) as the equivalent Young’s modulus.

Dramatic changes in grain fibre patterns have been demonstrated during early infancy—from clearly visible in newborns (Coats and Margulies 2006; Holck 2005; McPherson and Kriewall 1980) [an illustrative picture shown in Fig. 3] to almost invisible already in 6-month-olds (Margulies and Thibault 2000). The anisotropy ratio further decreases to 1.25 in 6-year-olds (Kriewall 1982) and becomes isotropic in adults (McElhaneyet al. 1970). Meanwhile, the skull bone gets stiffer with age, ranging from a few hundred MPa in infants (Coats and Margulies 2006) up to several GPa in 6-year-olds (Davis et al. 2012). Therefore, material modelling for infant skull at different ages should reflect the two biological growth factors, decrease with anisotropy and an increase in stiffness. Here, we present a new approach considering both factors, including the following steps:

1. Anisotropy ratio as a function of age [$f_{\mathrm{anisotropy}} ({\mathrm{age}})$] is obtained by fitting measured data reported in Kriewall (1982) from 16 newborns and a 6-year-old, together with a ratio of 1.0 in adult (assume 18 years old). A 2-term exponential function—one of the typical functions to describe biological growth (e.g. Savageau 1980)—is used, and the fitted curve is plotted in Fig. 4a and governed by the equation:
  2 $$\begin{aligned} f_{\mathrm{anistropy}} \left( {\mathrm{age}} \right)= & {} 0.9071\times \hbox {e}^{-0.3017\times \mathrm{age}}\nonumber \\&+\,1.398\times \hbox {e}^{-0.00155\times \mathrm{age}} \end{aligned}$$

**Fig. 3:**
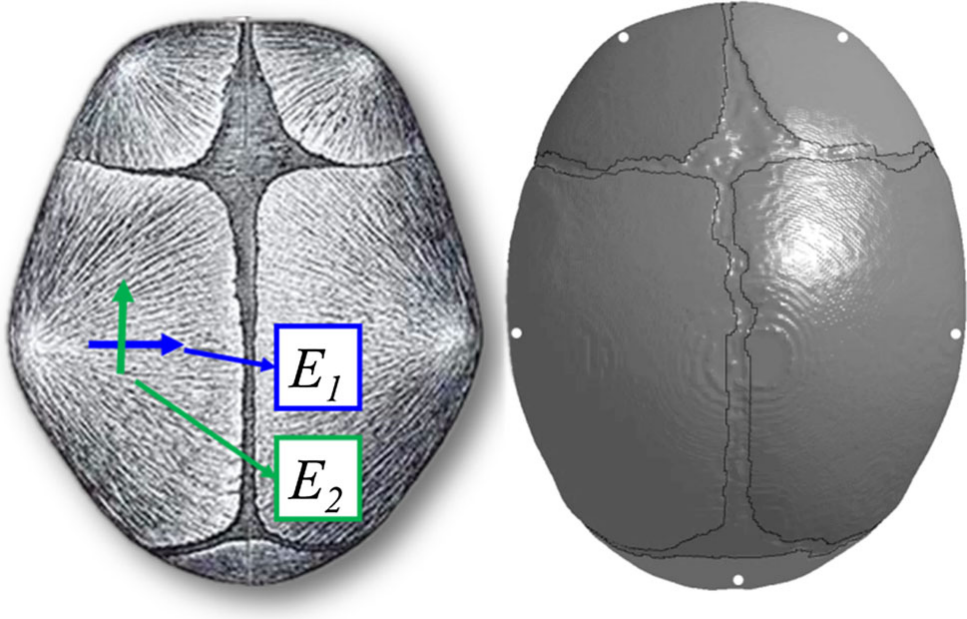
Illustration of the pronounced fibre orientation in infant skull bone, radiating from the ossification centres of each bone plate (*left*) [image adapted from Gray (1918)], and the ossification centres (indicated by *white dots*) assigned in the models according to the anatomical positions illustrated with the 5 M model (*right*)
2. $E_1$ is then calculated as $E_1 =E_2 \times f_{\mathrm{anistropy}} ({\mathrm{age}})$, with the measured values of $E_2$ from Coats and Margulies (2006). $E_1$ and $E_2$ as a function of age are obtained by fitting the calculated $E_1$ and measured $E_2$ from Coats and Margulies (2006) using a piecewise spline function, constrained to be a monotonic increasing and concave down function. The same procedure is done for parietal and occipital bone, respectively (Fig. 4b).

**Fig. 4:**
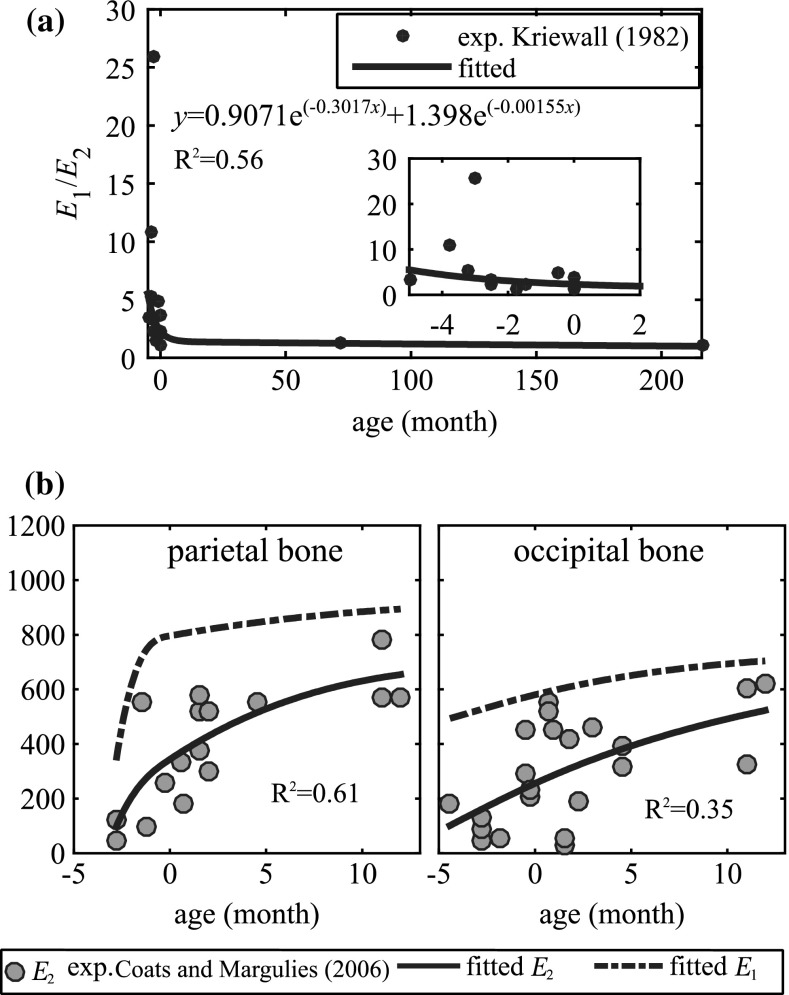
Process for the development of a material model for $E_1$ and $E_2$ considering both increase in stiffness and decrease in anisotropy with age. Gestation age of 40 weeks is defined as 0 month old, and a negative age represents a pretermed subject

Coefficient of determination $R^{2}$ is used to characterize the goodness of fit, which varies from 0 to 1, and a value of 1 indicates a perfect fit. The relatively low values of $R^{2}$ are due to the inherent variability of skull specimens from different infant subjects. The predicted anisotropy ratio for a newborn of 40-week gestational age is 2.31 from the above-fitted function, close to the mean value of 2.29 calculated from the data of four foetal calvaria at the same gestational age reported in Kriewall (1982). For a 6YO (72 M) and adult (18-year-olds), the predicted anisotropy ratios are 1.25 and 1.0, respectively, agreeing well with the reported value of 1.25 in Kriewall (1982), and isotropy of skull bone in adults. The derived skull bone elastic modulus for the newborn, 5 and 9 M, together with the material parameters for other tissues, are presented in Table 1. The properties of frontal bone are assumed to be the same as parietal bone.

After the parameters of $E_1$ and $E_2$ are determined, orthotropic elastic model can be implemented to describe the mechanical properties of the infant skull bone with fibres radiating from the ossification centres of each bone plate estimated according to their anatomical locations (Fig. 3). The orthotropic elastic material is represented using nine elastic constants including three Young’s moduli $E_1, E_2, E_3$, three Poisson’s ratios $\nu _{12},\, \nu _{13},\, \nu _{23}$ and three shear moduli $G_{12}, G_{23}, G_{31}$ (e.g. Robert 1998), where 1, 2 and 3 refer to the parallel-to-fibre, perpendicular-to-fibre and through-thickness directions, respectively. $E_3$ is assumed to be equivalent to $E_2$. Poisson’s ratio is assumed to be the same as in an adult ($\nu _{12} =\nu _{13} =0.22$, $\nu _{23} =0.19$) (McElhaneyet al. 1970), as with previous infant head models (Coats et al. 2007).

### Evaluation of model performance

The performance of the models is assessed by comparing the model predictions with those from cadaveric head tests reported in Loyd (2011), including both the 30-cm drop tests and the compression tests. The experimental tests included one 5 M, one 9 M and six newborn specimens. The developed FE models of the 5 and 9 M are compared with the tests of the same ages. For the newborns, although six specimens were tested, the exact impact angles were reported for only one specimen, which is chosen for model comparison for both the drop and compression tests. The specimen IDs and head sizes which the developed FE models are compared with are listed in “Appendix 1”.

**Fig. 5:**
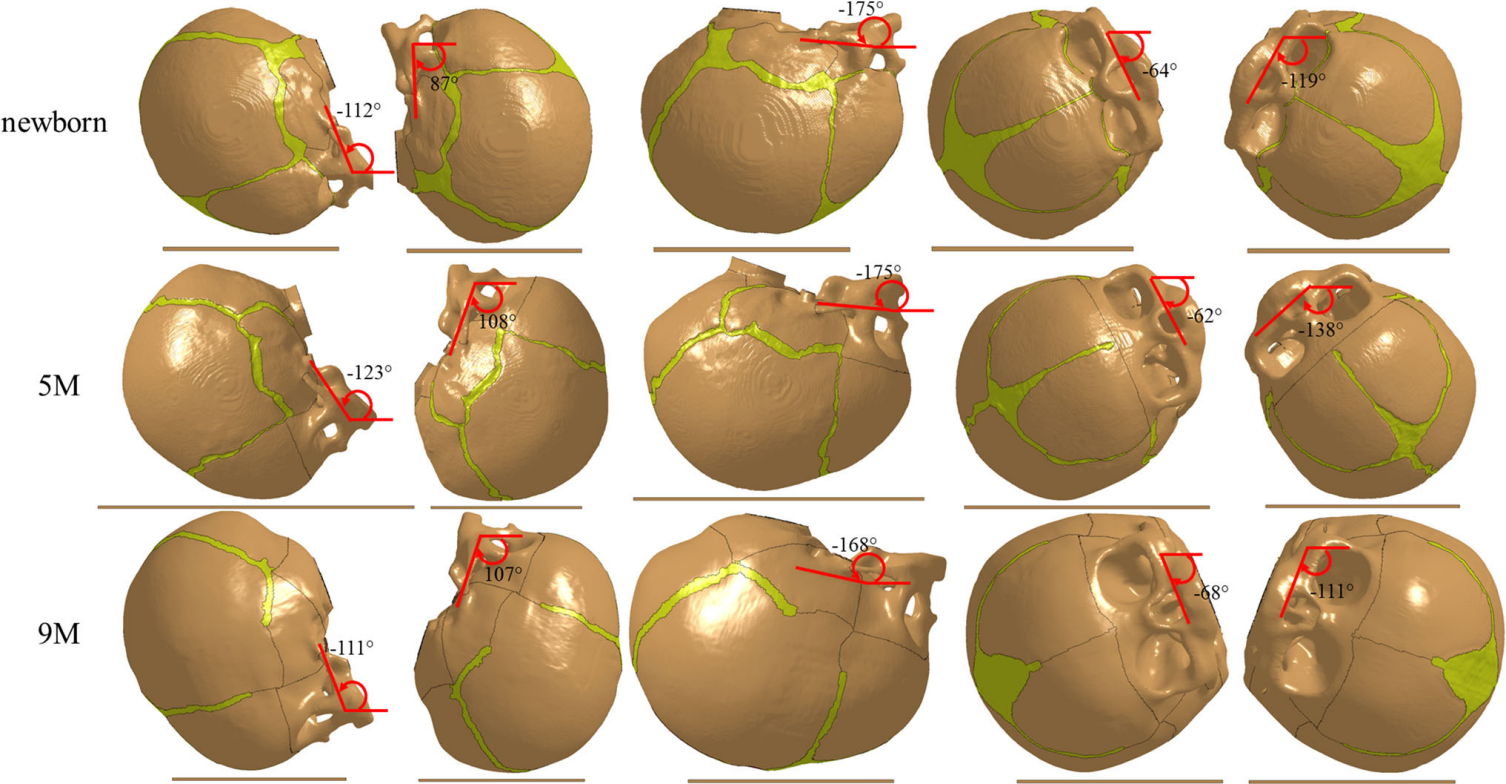
Simulations of forehead, occiput, vertex, and *right* and *left* parietal impact (*left* to *right*), with the impact angles illustrated for the newborn (*upper row*), 5 M (*middle row*) and the 9 M (*lower row*). The *scalp* is not shown

For the drop simulations at the five different impact locations, special care is taken to rotate the models to the same impact angle as in the experiments, as illustrated in Fig. 5. The signs for the angles follow the same definition as Loyd (2011). The impact angles for the right and left parietal impacts are missing for the 5 M in the experiment; therefore, estimated angles are used which are inferred from other age groups under the same impact locations. The end of the foramen magnum is fixed in the model to mimic the sealed end in the experiment. In the drop tests, an initial velocity is prescribed to all components of the infant head model, corresponding to the ideal free fall velocity from 30 cm height. No initial intracranial pressure is added in the model prior to the impact simulation, and the initial strain in the sutures is zero. Similarly, the head acceleration is calculated by dividing the impact force by head mass.

**Fig. 6:**
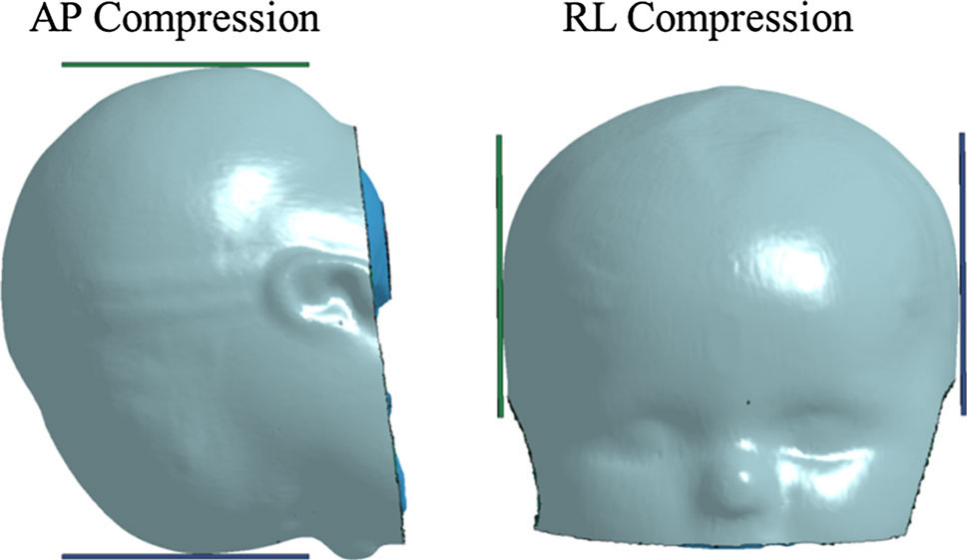
Simulations of AP and RL compression tests, illustrated with the 9 M model

The same setup is used in the compression simulations as in the experiments in both the anterior–posteriors (AP) and right–left (RL) directions (Fig. 6): the head is compressed along the Frankfort plane; the ears are removed for the RL compression to allow the plate to directly contact the head; and the end of the foramen magnum is left free in order to mimic the experimental apparatus, which used a gauze to loosely fix the cranial content during compression. In the compression simulations, one plate is fixed and the other is moving at a velocity calculated to achieve a compression rate of 0.3/s taking the head sizes into account (head length for AP compression and head width for RL compression).

The performance of the models is evaluated using correlation score (CS) to assess the agreement between the model prediction and the measured acceleration–time impact curves in terms of phase (*N*-phase), amplitude (*N*-amp) and shape (*N*-shape). The technical details have been published previously, see, e.g. Kimpara et al. (2006), but are also provided in “Appendix 3” for completeness. CS values range from 0 to 100, with values between $86\le \hbox {CS}<100$ classified as *excellent* according to a biofidelity rating (De Lange et al. 2005).

## Results

### Predictive performance of the model

**Fig. 7:**
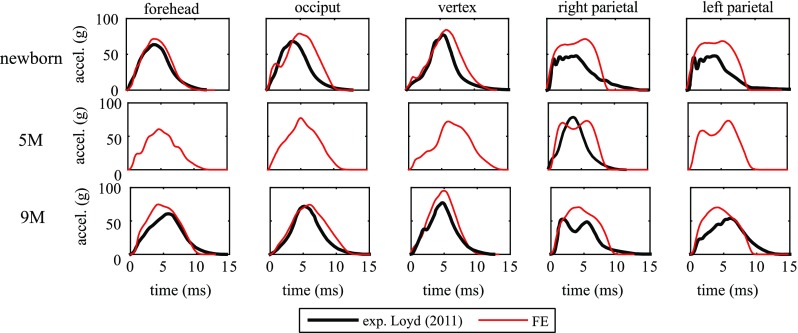
Simulated and experimental head acceleration–time curves for the impacts at five different locations for the newborn (*upper row*), 5 M (*middle row*) and the 9 M (*lower row*) with a drop height of 30 cm

**Fig. 8:**
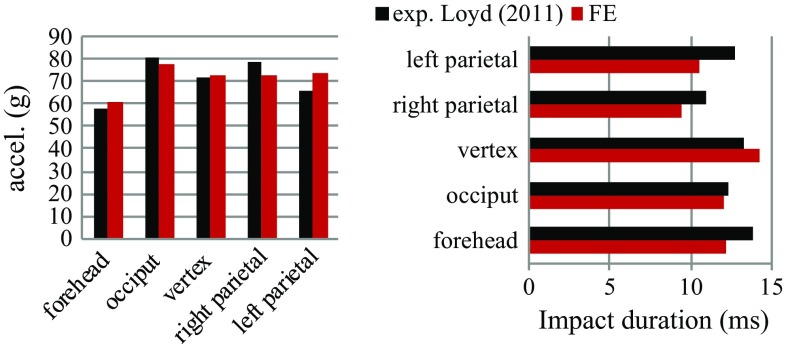
Simulated and experimental peak head acceleration (*left*) and impact duration (*right*) at different impact locations for the 5 M head model with a drop height of 30 cm

The acceleration–time impact curves generally correlate well with the measurements (Fig. 7), showing the expected curve characteristics at different impact locations (correlation score all above 86, see Table 2). The newborn parietal impact tests show different curve characteristics compared to other impact locations, which are well captured by the model although a difference is observed in the peak. The actual impact angle for the 5 M right impact used in the experiment is not available, making it impossible to use the same angle in the simulation. Thus, the discrepancy between the simulated curve and the experimental curve could be explained by the potential difference in the impact angle (see Sect. 3.4 for details). Further, note that the experimental data for the 5-month-olds head from Loyd (2011) presented in Figs. 7 and 8 were taken after the skull had fractured in the 15 cm right parietal impact.

For the 5-M experimental drop tests, the entire acceleration–time impact curve for a right parietal is reported (Fig. 7); for other four impact locations, only the peak acceleration and impact duration are reported (Loyd 2011), which are compared with the model predictions. Both the predicted peak acceleration and impact duration agree well with the experimental findings (Fig. 8).

Table 2 lists the correlation scores between the simulated and experimental acceleration–time curves at different impact positions. The CS values are calculated based on the acceleration–time curves sampled at a time interval of 0.2 ms. All the models have achieved an “*excellent*” performance in the drop tests according to a fidelity rating.

**Fig. 9:**
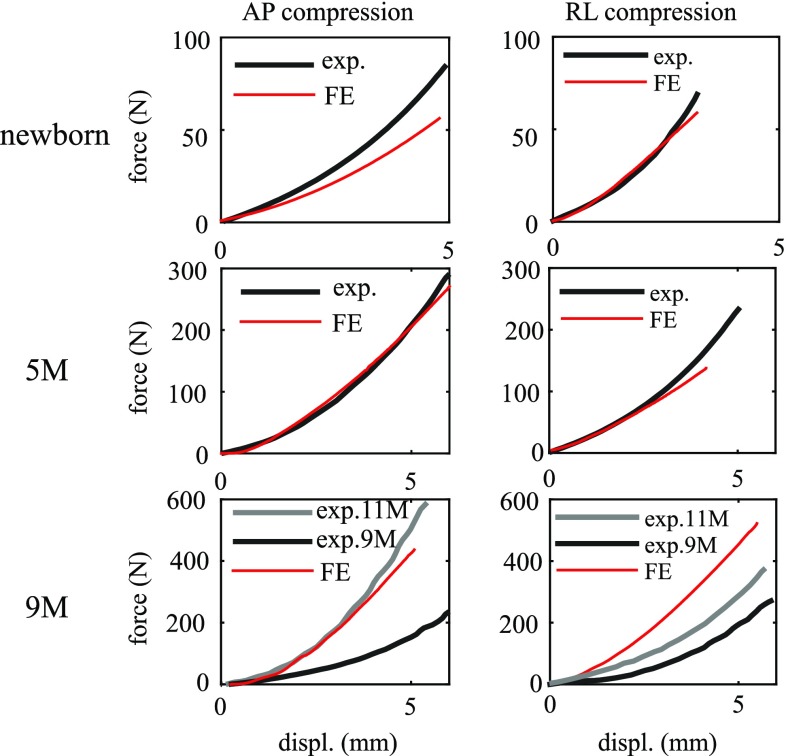
Simulated force–deflection curves for the newborn (*upper row*), 5 M (*middle row*) are comparable with the experimental data from the same age reported in Loyd (2011) with specimen IDs of P13F and P12M, respectively. For the 9 M (*lower row*), the experimental curves for both the 9 M (P14M) and 11 M (P15F) are presented

For the compression tests, the simulated force–deflection curves all exhibit an increased stiffness at larger displacements (Fig. 9), consistent with the experimental findings in Loyd (2011). The compression curves predicted from the newborn and 5 M model are comparable with the experimental data. For the newborn, the AP and RL compression curves from only one specimen (P13F) are chosen for comparison; it is, however, representative of all the tested newborn data [the compression curves from P13F lie between the curves of all other tested newborn specimens reported in Loyd (2011)]. The 9 M head model is stiffer than the 9 M cadaver head in the experiment, resulting a larger force for the same displacement. Note that the 9 M is even weaker than the 5 M in the experiments (the large-sized anterior fontanelle of this specimen might offer an explanation, see Discussion). The experimental compression curve for an 11 M specimen is therefore also plotted for comparison.

**Table 2:** Summary of CS values for the acceleration–time curves impacted at various locations

	$\hbox {CS}_{N\mathrm{-phase}}$	$\hbox {CS}_{ N\mathrm{-amp}}$	$\hbox {CS}_{N\mathrm{-shape}}$	Average
*Newborn*				
Forehead	98.98	99.19	99.44	99.20
Occiput	99.42	97.32	91.17	95.97
Vertex	98.89	97.95	91.25	96.03
Right parietal	99.40	90.14	96.71	95.41
Left parietal	99.73	88.65	96.50	94.96
*5 M*				
Right parietal	97.54	97.93	94.33	96.60
*9 M*				
Forehead	93.08	96.75	93.95	94.60
Occiput	99.37	98.55	98.40	98.77
Vertex	99.09	96.43	99.77	98.43
Right parietal	99.52	92.87	96.74	96.38
Left parietal	87.79	95.32	91.91	91.67

Note the 5 M model crashes in the RL compression test before it reaches the maximum displacement as in the experiment, making it impossible to compare with the entire experimental curve. Under compression, CSF is extruded outside of the cranial space, as the end of the foramen magnum is left free in order to mimic the experimental apparatus (see Sect. 2.4), which in turn causes a large distortion in the CSF mesh. The model crashes due to the inherent limitation of Lagrange mesh to handle large mesh distortions. Therefore, only part of the curve is compared with the experiment.

### Head deformation and impact surface area

**Fig. 10:**
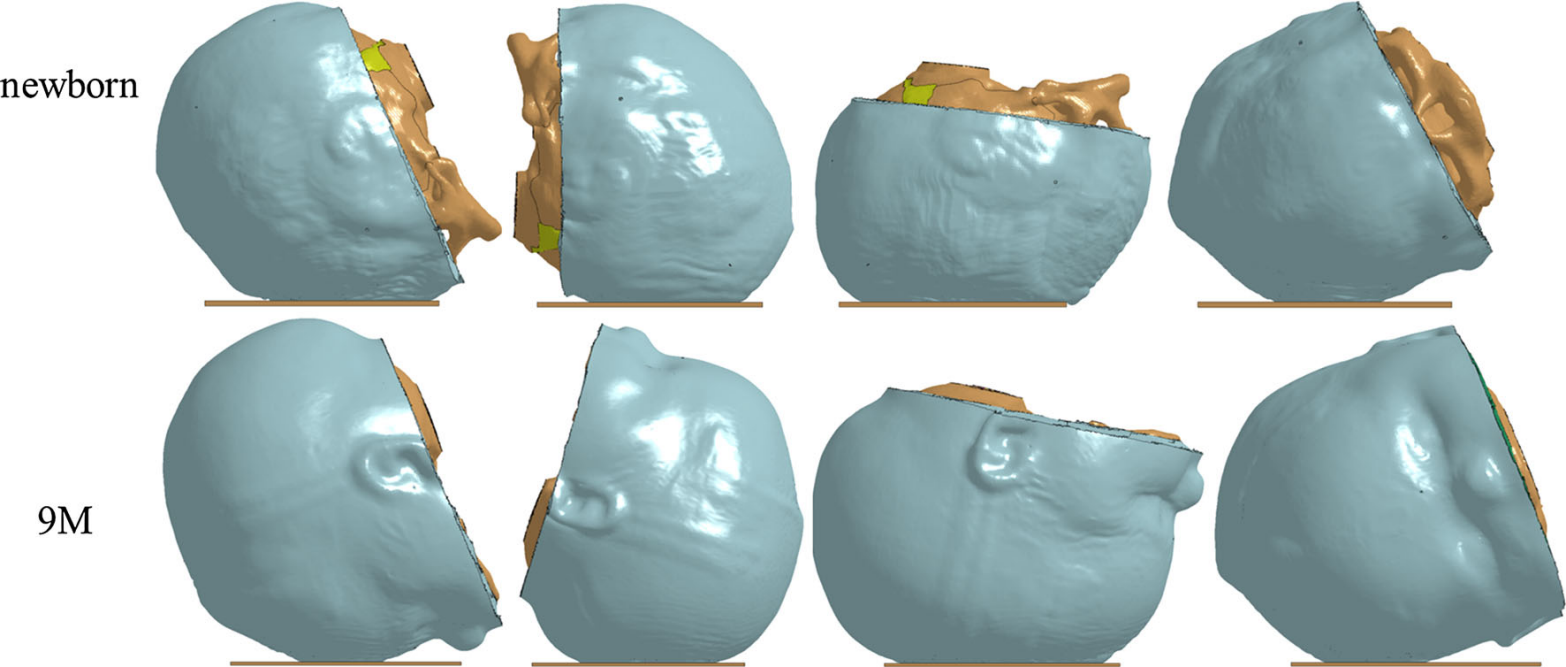
Profound head deformations during impact illustrated by the newborn (*upper row*) and the 9 M model (*lower row*). The images are captured when the head deformation reaches its maximum. From left to right: forehead, occiput, vertex and right parietal impact. Additional animations showing the dynamic impact response are provided as supplemental videos

The simulations reveal profound deformations of the infant heads under a 30-cm drop impact, as illustrated with the newborn and the 9 M model (Fig. 10). The vertex impact generally leads to a largest head deformation, followed by the occiput, then forehead and finally the parietal impact, which holds in all the three models. In particular, for a vertex impact, the newborn head deforms 13.2% (11.2 mm) along the impact direction, and the value changes to 10.48 (12.6) and 7.9% (10.2 mm) for the 5 and 9 M, respectively. The younger the age group, the larger percentage the head deforms, despite the absolute values do not follow this trend.

The paucity of experimental data makes it difficult to assess whether the predicted head deformations are reasonable. But a relevant measure—the impact surface area, has been reported in Loyd (2011) obtained by a pressure sensitive film placed on top of the impactor, which is compared with the model prediction (Table 3). The simulation shows that a vertex impact in general leads to the largest impact surface area, followed by occiput, and then forehead impact, the same is seen in the experiments. Considering the head deformations shown in Fig. 10, it appears that an impact location with larger head deformation also lead to a larger impact surface area. Further, both the experimental and the simulated impact surface area suggest an overall increase with age, which is consistent with animal drop tests using infant porcine specimens (Baumer et al. 2010).

**Table 3:** Impact surface area ($\hbox {cm}^{2})$ at different impact locations from the simulation compared with the experimental data reported in Loyd (2011)

	Forehead	Occiput	Vertex	Right parietal	Left parietal
*Newborn*					
Simulation	9.44	9.50	22.9	5.86	6.31
exp. P13F	8.4	8.6	22.2	–	–
*5 M*					
Simulation	12.2	19.0	18.8	6.24	10.3
exp. P12M	–	–	–	–	–
*9 M*					
Simulation	12.6	22.3	36.4	11.4	8.91
exp. P14M	17.5	25.9	34.4	–	–

“–” indicates experimental data not provided in Loyd (2011)

### $1{\mathrm{st}}$ principal Green–Lagrange strain in the skull bone during impact

**Fig. 11:**
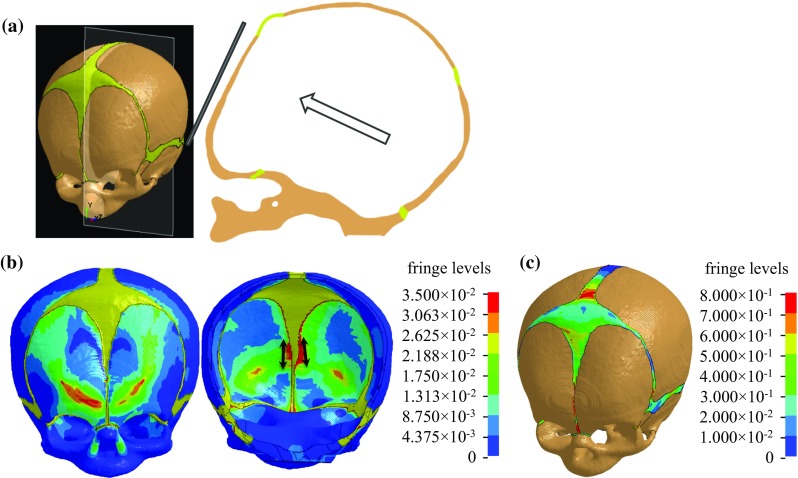
**a** Sagittal plane of the newborn skull deformation under a forehead impact when the deformation reaches its maximum. **b** The $1{\mathrm{st}}$ principal Green–Lagrange strain in the skull viewed from outside (*left*) and inside (*right*), the arrows indicate the direction of the $1{\mathrm{st}}$ principal Green–Lagrange strain which is approximately parallel to the skull edges. **c** The $1{\mathrm{st}}$ principal Green–Lagrange strain in the suture in the newborn forehead impact

The deformation pattern, the $1{\mathrm{st}}$ principal Green–Lagrange strain of the newborn skull and suture under a forehead impact are presented in Fig. 11. The frontal bone plates deform to a flat shape following the impactor surface (Fig. 11a). Further, the $1{\mathrm{st}}$ principal Green–Lagrange strain in the skull shows larger values at the interfaces between the skull and the sutures/fontanelles, compared with other areas, and the values are larger at the inner table of the skull than the outer table adjacent to the interfaces (Fig. 11b). The direction of the $1{\mathrm{st}}$ principal strain, representing the maximum stretching direction, is approximately parallel to the skull edges at the interfaces (Fig. 11b, right). The $1{\mathrm{st}}$ principal Green–Lagrange strain in the suture rises up to 0.8 (Fig. 11c).

### Influence of impact angle

The influence of impact angle is investigated using an occiput, right parietal impact with the newborn model, and a right parietal impact with the 5 M model. For the baseline newborn occiput impact, a secondary peak is observed in the simulated curve but not experimentally (Fig. 7). Therefore, the newborn head model is rotated from its baseline of $87^{\circ }$ to $102^{\circ }$ to see whether the inconsistency could resolve by a different impact angle. For the newborn right parietal impact, the model is rotated from its baseline of ${-}64^{\circ }$ to a pure lateral impact of ${-}90^{\circ }$, which appears to be an intuitive angle when referring to a parietal impact, and seems to be used previously [shown in a schematic figure by Li et al. (2013a)]. It is also noted that the simulated acceleration–time curve in the 5 M right parietal impact with an estimated impact angle is quite different from the experimental curve (see Fig. 7). Thus, an impact angle of ${-}90^{\circ }$ is further simulated to test whether it is possible to produce a similar curve as in the experiment.

**Fig. 12:**
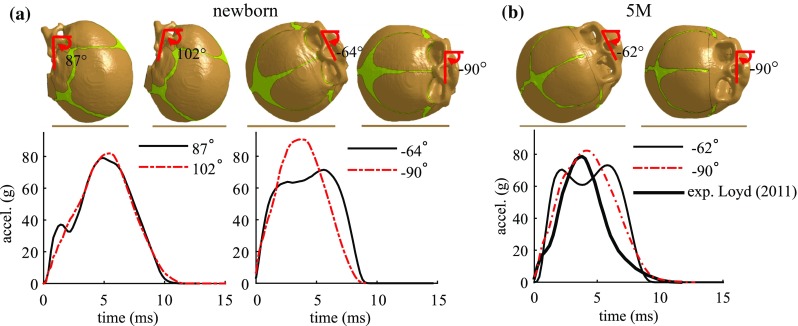
**a** Influence of impact angle in an occiput (*left*) and right parietal (*right*) impact for the newborn model. **b** Influence of impact angle in a right parietal impact for the 5 M model

The $102^{\circ }$ occipital impact leads to a similar impact curve as the baseline of $87^{\circ }$, but the secondary peak in the curve disappears, and becomes consistent with the experiment. While in the right parietal impact, a profound change is seen in both the peak acceleration and the curve characteristics when rotating the model to a ${-}90^{\circ }$. For the 5 M right parietal impact, when rotating the model to ${-}90^{\circ }$, the impact curve pattern becomes consistent with the experimental curve, meaning that an angle close to ${-}90^{\circ }$ could have been used in the experiment instead of the estimated angle of ${-}62^{\circ }$ in the baseline model shown in Fig. 5. Note that the 5-month-olds head from Loyd (2011) fractured on the 15 cm right parietal impact, and thus, the subsequent 30 cm impact acceleration maybe be lower than if without fracture, which to some extent explains the slightly lower peak acceleration in the experiment than the model (Fig. 12b).

For parietal impacts, besides the angle viewed from the front of the face (see Fig. 5), there is a second angle that runs along the front to back of the head needs to be considered, but it is not reported in Loyd (2011). This may also have an effect on the response. The profound influence of impact angle for a parietal impact is consistent with the experimental data from Loyd (2011). It appears that the more horizontal the head impacted at parietal, the more likely the impact curve presents with a single and a higher peak, as shown in both the newborn and 5 M model (Fig. 12).

### Influence of constitutive modelling of soft tissues

To compare with existing infant head models, parametric studies using the same linear elastic models for the scalp, suture, dura are performed with the newborn model. A forehead impact is chosen, as well as right parietal impact due to its special impact curve characteristics. A model with the sutures removed is also studied. The baseline model refers to the newborn model with the nonlinearly elastic material parameters presented in Table 1. Linear elastic models in the parametric study adopt linear elastic constants that have been used in the existing infant head models (Coats et al. 2007; Roth et al. 2010; Li et al. 2013b, 2016) (Table 4).

**Table 4:** Parameters for suture, scalp, dura and skull used in the parametric study

Baseline model	Nonlinear elastic suture, scalp, and dura, see Table 1
Suture	Linear elastic suture, $E=8.1\,\hbox {MPa}$, $\nu =0.49$
	No suture
Scalp	Linear elastic scalp, $E=16.7\,\hbox {MPa}$, $\nu =0.42$
Dura	Linear elastic dura, $E=31.5\,\hbox {MPa}$, $\nu =0.45$

#### Forehead impact

**Fig. 13:**
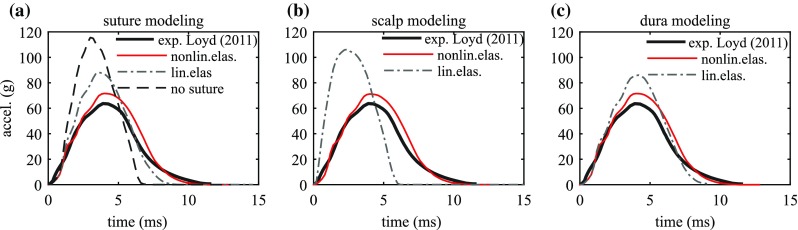
Influence of constitutive modelling of the scalp, suture, dura and skull on the acceleration–time impact curve for the newborn forehead impact

For a forehead impact, a linear elastic suture model increases the peak acceleration by 22.4% compared to the nonlinear elastic suture model (Fig. 13a). A no-suture model leads to a substantial increase in the peak acceleration by 61.3%, as well as an increased von Mises stress in the skull bone by 55.6%.

A linear elastic scalp increases the peak acceleration by 49.2%; however, it decreases the peak von Mises stress in the skull bone by 22.4% (Fig. 13b). This observation can be explained by the stiffening effect of the linear elastic scalp, which allows less skull bending during impact compared to the baseline scalp model. It also results in a profound decrease in both the impact duration and impact area (Fig. 13b; Table 5). The linear elastic scalp fails to produce a realistic impact curve, unlike the nonlinear elastic scalp model. Although only a small change is seen in the peak von Mises stress in the skull using the linear elastic dura mater, an increase of 20.1% is found in the peak acceleration compared to a nonlinear elastic dura (Fig. 13c).

**Table 5:** Results of the parametric study of a 30-cm fall for the newborn forehead impact

	Peak von Mises stress (MPa) in the skull bone	Peak acceleration (g)	Max contact area $\left( \hbox {cm}^{2}\right)$	Impact duration (ms)
*Baseline*				
Nonlinear elastic suture, scalp, and dura	21.11	71.66	9.44	11.19
*Suture*				
Linear elastic	23.55	88.14	10.4	9.02
No suture	32.85	115.58	10.8	7.0
*Scalp*				
Linear elastic	16.39	106.9	2.03	6.08
*Dura*				
Linear elastic	22.31	86.09	10.6	9.4

#### Right parietal impact

**Fig. 14:**
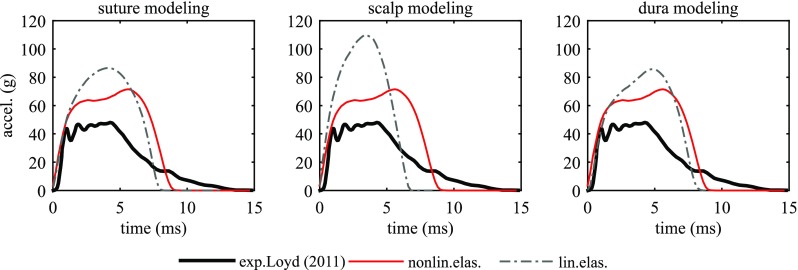
Influence of linear elastic modelling of suture (*left*), scalp (*middle*) and dura (*right*) for the newborn right parietal impact

For a right parietal impact, the simulations show that any of the three tissues using linear elastic model fail to reproduce the special curve characteristics seen in the experiment and scalp has the largest influence (Fig. 14).

## Discussion

In this study, we present new nonlinear constitutive laws of soft tissues for infant head modelling, which lead to more realistic performance compared with the linear elastic models that are widely used in the existing infant head models. Note the comparison between nonlinear models and linear elastic models includes both material models and their constants throughout the study. We develop a new approach for obtaining age-dependent skull bone orthotropic parameters. Subject-specific head models of a newborn, 5 and 9 M incorporating above efforts on constitutive modelling produce global head impact response that agree well with experimental data for both drop and compression tests. The improved nonlinear models for the suture, scalp and dura, together with an accurate impact angle, enable the models to predict acceleration–time curves and impact surface area that are comparable with experimental findings at various impact locations, as well as force–deflection curves in compression simulations. Particularly, the acceleration–time curve with special characteristics for parietal impact is predicted for the first time. The simulations show profound deformation of infant head resulting a large $1{\mathrm{st}}$ principal Green–Lagrange strain at the interfaces between the bone and the sutures/fontanelles, unique to the infant head compared to older children and adults where the skull bones are fused. Impact angle has a profound influence on both the global head impact kinetics and skull injury metrics, especially for parietal impacts. Thus, impact angle should be considered for a true and realistic validation of infant FE head models, as well as evaluating the risk of infant skull fractures.

Compared with the baseline models with nonlinear elastic materials for suture, scalp and dura mater, the head models using linear elastic models for the soft tissues are too stiff since the linear elastic models with Young’s modulus determined at the linear region do not allow accounting the less stiff stage due to uncramping in soft tissues. Especially the linear elastic scalp model produced a much larger peak acceleration compared with the experiments for a parietal impact as illustrated with the newborn model (Fig. 14). Note previous models using the same linear elastic constants for scalp were able to produce peak accelerations comparable with experimental data (Li et al. 2013b; Roth et al. 2010). In these models, the skull bone was modelled as an isotropic material taking the Young’s modulus measured at the perpendicular-to-fibre direction ($E_2$) from (Coats and Margulies 2006) as the equivalent value, causing the skull bone softer than in reality. Particularly, the skull bone stiffness has been shown to be a most dominant factor influencing the peak acceleration (Li et al. 2013a, b).

The limitations of linear elastic models are further revealed in parietal impacts—any of the three tissues using linear elastic material fails to produce a curve with special characteristics for a parietal impact. Unlike forehead, occiput and vertex impacts, which often lead to typical single-peak impact curves, parietal impacts can result impact curves with different characteristics as shown both from experiments and from the simulations (Fig. 7). The simulation results show that both the existence of the unique curve pattern in parietal impacts, and to which extent it appears are closely affected by the way in which the bones are allowed to move relative to the suture. This relative motion in turn is largely affected by material properties of scalp enclosing the head, dura mater attached to the skull, and the suture connecting the bone plates. The baseline model with nonlinear elastic material for all the three components is soft at small strains, allowing a larger movement between the skull bones (see additional animations of the newborn right parietal impact), which enables the model to produce the parietal impact curve with special characteristics. A similar relative motion between the bones is also seen in the 5 M right parietal impact simulation with a ${-}62^{\circ }$ angle, resulting in a special curve pattern curve as shown in Fig. 7. While any of the three soft tissues modelled with linear elastic models will cause the head to behave too stiffly and allows less the relative motion of bones, resulting acceleration–time curves with a single peak (Fig. 14). It is noteworthy that both the experimental and the simulation show a parietal impact can also lead to a single-peak curve when the model lies more horizontally approaching to ${-}90^{\circ }$ (Fig. 12). A closer examination of the simulation shows under such impact angle, the lower skull bone got contact with the impactor and allows less relative motion between the skull bones. The head models using nonlinear soft tissue material models in this study are capable to produce both types of parietal impact curves as shown with the newborn and the 5 M model (Fig. 12).

The proposed approach for obtaining $E_1$ by scaling with fitted anisotropy ratios describes the rapid decrease in anisotropy during the early infancy, reaches nearly isotropy in 6-year-olds and further approaches to isotropy in adults. Note the only existing direction-specific infant cranial bone data ($E_1$ and $E_2$) reported in the literature were performed on foetal specimens from 20- to 42-week gestational age (Kriewall 1982; McPherson and Kriewall 1980), which does not apply to older specimens due to the vast changes in grain fibre patterns during early infancy. Until new experimental data are available, the approach presented in this study allows infant head models to incorporate the direction-specific cranial bone properties at different ages from premature to 6 years old.

Impact angle has a profound influence on both the peak acceleration and the curve characteristics for parietal impact, but less influence is found for an occiput impact, as shown with the newborn model (Fig. 12a). Experimental studies on paediatric head impacts are very limited; Prange et al. (2004) and Loyd (2011) are the only studies performed quantitative measurements so far. Unfortunately, the actual impact angles for some of the drop impacts were not reported, which raises a question—what impact angle should be used in the model when validating against such experimental data? Especially the impact angles can differ a lot for the same impact location (see Loyd 2011). While it may seem obvious that impact angles affect the head responses, a quantitative analysis on the influences of impact angle is necessary. For impact locations where the impact angle has a minimal influence [e.g. occiput impact for the newborn (Fig. 12)], using a reasonably assumed impact angle in the model could be acceptable. However, for parietal impacts where impact angles are critically influencing the head responses, one has to be cautious when using such data for validation if the impact angles in the experiments are unknown. The influences of impact angle at other impact locations, and for other age groups are yet to be determined to better guide using these precious experimental data for validating biomechanical models.

Gurdjian et al. (1950), using a stress coat technique, found that adult skull, when strikes a flat surface, flattens out at the point of impact to conform to the shape of the surface against which it impacts, while the peripheral areas are bent outward forming the typical “out-bending” area, which skull fractures usually initiates. Here we show that infant skull deforms to a flat shape following the impactor surface (Fig. 11) and no “out-bending” area is formed. The presence of suture and fontanelle is expected to play an important role causing such a difference in the skull deformation pattern compared with adults’ head. Consequently, a large $1{\mathrm{st}}$ principal Green–Lagrange strain (or stretch) is observed at the interface between the skull bones and the suture/fontanelle, suggesting that infant skull fractures are likely to initiate from the interfaces before the fractures propagate to other areas. This observation is consistent with experimental findings using infant porcine skull, where fractures initiated at bone–suture interfaces (Baumer et al. 2010). While in the studies using human PMHS by Weber (1984, 1985) only the fracture patterns were depicted and no discussion on the initiation of fractures was provided.

The simulations reveal profound head deformation already at a drop height of 30 cm. Then can the massive deformation cause rupturing of the bridging veins leading to subdural haematomas in infants? The answer to this question remains unclear, although Weber (1984) reported that some drops from 82 cm using PMHS indeed resulted in small haemorrhages in the area of the bridging veins.

The geometrical meshes for the newborn, 5 and 9 M head models are created from CT images of similar ages of a newborn, 4 and 8 M. To cope with the geometrical variations between the models and the cadaver heads which the models are compared with, the head models have been scaled to the head sizes in the experiment (see “Appendix 1” for details), and the same head mass is ensured by selecting a certain amount of scalp elements (see Methods). However, inherent morphological differences between the cadavers and the FE models can still exist, such as skull shape, thickness and material properties, even at the same age. In particular, fontanelle and suture sizes can vary widely in infants (Faix 1982). We notice that the 9 M specimen in the experiment has an extremely large fontanelle [shown a picture in an unrelated study, published by the same group (Mulroy et al. 2012)]. Coincidently, the CT image of the 8 M based on which the FE model is generated also has an exceptionally large anterior fontanelle, which is comparable with the 9 M specimen. Thus, the drop and compression test results from both the experiment and the simulation presented in Figs. 7 and 9 maybe more compliant compared with heads with normal sized fontanelle at the same age, while the anterior fontanelle sizes for the newborn and the 5 M are within normal range according to the data reported in Popich and Smith (1972).

For the newborns, the material properties and suture/fontanelle sizes depend heavily on the gestational ages. The newborn model is generated from CT image of foetus at 39-week gestation, while the cadaver head in the experiment that the developed model is compared with is at 34-week gestation (Loyd 2011). Thus, the skull bone material properties (anisotropy ratio and elastic constants) for the newborn model are calculated correspond to 34-week gestation. Although a scaling factor of 0.8 is applied to the original newborn FE model of a 39-week gestation to account for a smaller sized head at 34-week gestation (see “Appendix 1”), the suture and fontanelle sizes may still be smaller than at 34-week gestations age. Because the relative size of sutures/fontanelles is not accounted during this global scaling, this may to some extent explain the higher accelerations in the simulations than in the experiments. Further, in the current models, no failure in the skull is included, as fractures are unlikely to occur at a drop height of 30 cm (Loyd 2011; Prange et al. 2004). Nevertheless, an enhanced skull model with failure can be readily implemented based on the current model to study infant skull fractures.

### Electronic supplementary material

Below is the link to the electronic supplementary material.

Supplementary material 1 (wmv 470 KB)

Supplementary material 2 (wmv 533 KB)

Supplementary material 3 (wmv 603 KB)

Supplementary material 4 (wmv 752 KB)

Supplementary material 5 (wmv 900 KB)

Supplementary material 6 (wmv 869 KB)

Supplementary material 7 (wmv 791 KB)

Supplementary material 8 (wmv 713 KB)

## Figures and Tables

**Fig. 15:**
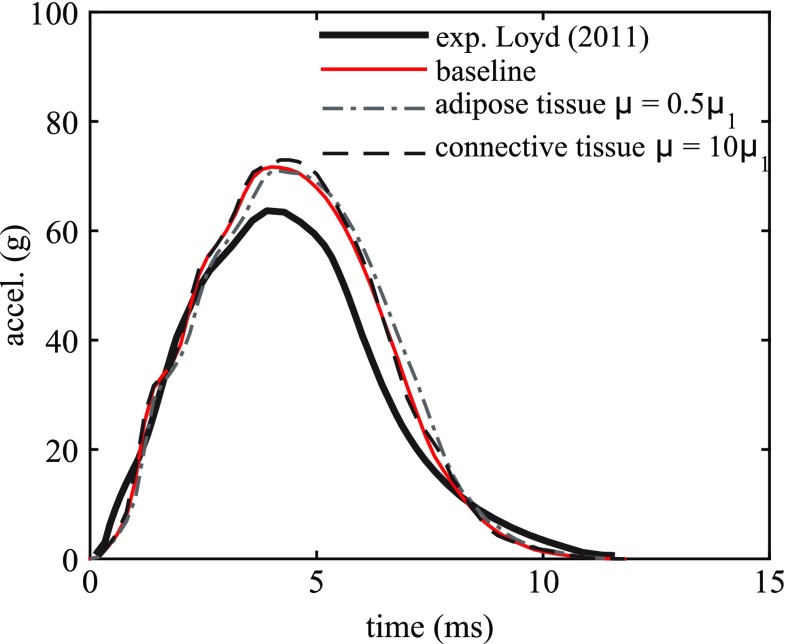
Simulated head acceleration–time curves for a newborn forehead impact with different scalp stiffness parameters, where $\mu _{1}$ is the Ogden parameter and the baseline values are listed in Table 1

## Appendix 1: Anatomical features of the generated FE head models

The sizes of the PMHS heads which the developed FE models are compared with are listed in Table 6; the sizes of the originally generated FE head models together with the factor used to scale the original FE head models to the PMHS heads are listed in Table 7.

**Table 6:** Head sizes of the newborn, 5 and 9 M specimens reported in Loyd (2011)

Specimen ID	Length (cm)	Width (cm)	Circumference (cm)	Characteristic length (CL) (cm)
Newborn (P13F)	10.7	7.2	29.7	47.6
5 M (P12M)	13.1	10.9	40.8	64.8
9 M (P14M)	14.8	12.0	46.5	73.3

**Table 7:** Head sizes of generated FE models and the scaling factor used to scale to the PMHS heads

Original FE model	Length (cm)	Width (cm)	Circumference (cm)	Characteristic length (CL) (cm)	Scaling factor based on CL
Newborn	12.8	10.2	37.0	60.0	0.8
5m	14.8	11.6	41.3	67.8	0.96
9m	15.6	12.8	45.2	73.7	0.99

The thickness of the scalp varies at different regions, being the thickest at forehead for infants and children ${<}$8 years old [see, e.g. a review article by Margulies and Coats (2013)]. The scalp thicknesses in the scaled FE models at impact site are measured manually along the impact direction (Table 8), which are in the range of literature data for specimens at similar ages reported by Young (1959).

**Table 8:** Thicknesses of scalp (in mm) measured at the impact sites in the FE models

	Forehead	Occiput	Vertex	Right parietal	Left parietal
Newborn	4.7	2.2	3.7	2.5	2.8
5 M	3.4	2.5	3.2	2.4	2.9
9 M	4.2	3.5	4.1	3.4	3.4

The thickness of sutures also varies at different regions. Soboleski et al. (1997) measured suture thicknesses in 50 neonates and infants between 0–5 months old, and the average thicknesses across all ages are reported to be $1.97\pm 0.54$, $1.88\pm 0.56$ and $2.49\pm 0.86\,\hbox {mm}$ for coronal, sagittal and lambdoid suture, respectively. Following the same approach, the thicknesses of sutures at the narrowest place are measured in the scaled FE head models and are listed in Table 9.

The thicknesses of sutures in the head models are in the range of the literature data by Soboleski et al. (1997), except the newborn suture thicknesses are below the lower limit, and the 9 M model coronal suture thicknesses are above the upper limit. Considering the average thicknesses reported in Soboleski et al. (1997) were based on specimens of 0–5 months old, the suture thicknesses of the newborn model scaled to 34-week gestation and the 9 M outside the reported range is considered reasonable.

## Appendix 2: Parametric study of scalp material constants

A newborn forehead impact is used to study the influence of scalp material constants on the acceleration–time curve. The results show that halving of the adipose tissue stiffness decreases the peak acceleration by 1.0%, and a 10 times stiffer outer layer increases the peak acceleration 1.8% (Fig. 15).

**Table 9:** Thicknesses of sutures (in mm) in the FE models

	Coronal	Saggital	Lambdoid
Newborn	1.2	1.3	1.1
5 M	2.4	2.6	2.0
9 M	2.6	2.3	3.1

## Appendix 3: Correlation score (CS) calculation

The error measures (*N*-phase, *N*-amp, *N*-shape) estimated by the NISE method are used to calculate a CS. CS values range from 0 to 100. Detailed equations are presented below.

To compute *N*-phase, *N*-amp, *N*-shape for two discrete time histories $X_i$ and $Y_i$, four correlation functions are defined and expressed as:

3 $$\begin{aligned} R_{xy} \left( 0 \right)= & {} \frac{1}{N}\sum \limits _{i=n}^N \left( {X_i Y_i } \right) \nonumber \\ R_{yy} \left( 0 \right)= & {} \frac{1}{N}\sum \limits _{i=n}^N \left( {Y_i Y_i } \right) \nonumber \\ R_{xx} \left( 0 \right)= & {} \frac{1}{N}\sum \limits _{i=n}^N \left( {X_i X_i } \right) \nonumber \\ R_{xy} \left( \tau \right) \hbox {max}= & {} \frac{1}{N-n}\sum \limits _{i=1}^{N-n} \left( {X_i Y_{i+n} } \right) \end{aligned}$$

where *N* is the number of data points in each curve, $\tau$ is the time delay that maximizes function $R_{xy} (\tau )$, *n* is the time shift index and is defined as $\tau$ divided by the constant time step. The portions of the NISE due to phase shift (*N*-phase), amplitude difference (*N*-amp) and shape difference (*N*-shape) are defined as:

4 $$\begin{aligned} N\hbox {-phase}= & {} \frac{2R_{xy} \left( \tau \right) \hbox {max}-2R_{xy} \left( 0 \right) }{R_{xx} \left( 0 \right) +R_{yy} \left( 0 \right) } \nonumber \\ N\hbox {-amp}= & {} \frac{R_{xy} \left( \tau \right) \hbox {max}}{\sqrt{R_{xx} \left( 0 \right) R_{yy} \left( 0 \right) }}-\frac{2R_{xy} \left( \tau \right) \hbox {max}}{R_{xx} \left( 0 \right) +R_{yy} \left( 0 \right) } \nonumber \\ N\hbox {-shape}= & {} 1-\frac{R_{xy} \left( \tau \right) \hbox {max}}{\sqrt{R_{xx} \left( 0 \right) R_{yy} \left( 0 \right) }} \end{aligned}$$

The CS is then defined as:

5 $$\begin{aligned} \hbox {CS}_{N\mathrm{-phase}}= & {} 100\left( {1-\left| {N\hbox {-phase}} \right| } \right) \nonumber \\ \hbox {CS}_{N\mathrm{-amp}}= & {} 100\left( {1-\left| {N\hbox {-amp}} \right| } \right) \nonumber \\ \hbox {CS}_{N\mathrm{-shape}}= & {} 100\left( {1-\left| {N\hbox {-shape}} \right| } \right) \end{aligned}$$
